# Analysis of Cricket Ball Type and Innings on State Level Cricket Batter’s Performance

**DOI:** 10.3389/fpsyg.2019.02347

**Published:** 2019-10-24

**Authors:** Jonathan Douglas Connor, Wade H. Sinclair, Anthony S. Leicht, Kenji Doma

**Affiliations:** Sport and Exercise Science, James Cook University, Townsville, QLD, Australia

**Keywords:** cricket batting, performance analysis, constraints, ecological dynamics, coaching

## Abstract

**Background:** The aim of this investigation was to compare the type of cricket balls utilized and innings on cricket batting performance in the First-Class Australian competition.

**Methods:** Batting performance measures of 43 state level cricket batters were collected from two seasons of the Sheffield shield tournament (*N* = 60 games) that incorporated both Kookaburra^™^ (*n* = 30 games) and Duke^™^ (*n* = 30 games) cricket balls.

**Results:** First-innings batting performances were significantly greater for the average number of runs scored (37.5 ± 13.4 vs. 31.2 ± 11.3), balls faced (60.7 ± 26.2 vs. 49.9 ± 23.6), boundary 4s (3.8 ± 1.9 vs. 2.9 ± 1.4), and boundary 6s (0.2 ± 0.3 vs. 0.1 ± 0.3) scored per game (*p* < 0.05), as well as centuries scored (5.74 ± 8.56 vs. 1.49 ± 5.14%) compared to second innings performances (*p* < 0.05). There were no differences for any batting performance measures as a result of ball type (*p* > 0.05). However, significantly more wickets were taken by pace bowlers during Duke^™^ ball games (85.0 ± 12.8 vs. 76.4 ± 13.9%), while relatively more wickets were taken by spin bowlers during Kookaburra^™^ ball games (14.2 ± 12.5 vs. 22.0 ± 14.1%; *p* < 0.05).

**Conclusions:** Cricket batting performance was comparable in games involving the Kookaburra^™^ or Duke^™^ ball. However, pace bowlers were more successful transferring their skill to the Duke^™^ ball, while spin bowlers were more successful with the KB^™^ ball. Subsequently, batters may be able to effectively adapt their movement technique, and transfer their skill to the Duke^™^ ball conditions. Future research is suggested to examine the influence of the cricket playing surface’s deterioration on cricket batter’s interceptive performance.

## Introduction

Cricket batting is a complex perceptual-motor skill that also involves overcoming capricious task demands and permutable constraints. One major task constraint thought to impact on batting performance is the physical properties of the ball (Mehta et al., 1983; Weissensteiner et al., 2011). The majority of cricket played internationally use different types of cricket balls depending on the home country. For example, the Australian cricket competition uses a Kookaburra™ (KB™) ball that has a reported wider and flatter seam compared with an English Duke™ ball, which is slightly smaller and has a more pronounced seam (Rundell, 2009). Anecdotally, the Duke™ ball has also been stated to retain its smooth polished surface (referred to as ball shine) better after repeated contacts with the ground and bat. Studies have indicated that seam prominence and ball shine are pertinent factors that can alter the aerodynamics of a ball such that it follows a curvilinear flight path (i.e., swing bowling) (Mehta, 1985; Alam et al., 2007). These physical properties have been shown to contribute to larger ball swing (i.e., curvilinear movement through the air), albeit judicious use of the ball seam position by fast bowlers (Mehta, 2005) and ideal ball properties are also necessary ingredients (Barton, 1982). While ball trajectory (i.e., linear versus curvilinear) has been reported to alter cricket batter’s gaze behavior (Sarpeshkar et al., 2017a) and kinematics (Sarpeshkar et al., 2017b), it is yet to be substantiated whether different ball types demonstrate different ball-flight characteristics, bowler performance and subsequent batting performance. This would seem particularly important, as the Australian domestic competition has introduced a Duke™ ball during the latter half of their First-Class competition, in an effort to help batters adapt to the ball used in English conditions. Evaluating the impact of ball type on batting performance would have a substantial impact on the acute and chronic preparation of skilled cricket batters.

Another unique factor thought to impact upon batting performance is the order of innings, or more specifically, game time. Batters must contend with the deterioration of the playing pitch surface, which occurs across multiple innings (Carré and Haake, 2000; James et al., 2005). Interestingly, the influence of a changing pitch surface on cricket batting movements and behavior has been scarcely investigated. In other interactive sports such as tennis, studies have reported that various ground conditions (i.e., clay vs. grass surfaces) can change tactical strategies (O’Donoghue and Ingram, 2001; O’Donoghue and Liddle, 2002), physiological demands (Pereira et al., 2016), and performance outcomes (Gillet et al., 2009; Reid et al., 2013). However, there has been limited to no empirical examination in relation to the various cricket specific skills. Cricket batting is particularly unique in that, as pitch surfaces change their physical properties due to repeated forceful contact of the ball and players running on the pitch, so can the ball trajectory spontaneously change when contacting uneven areas of the pitch (James et al., 2005). These changes in ball trajectory and bounce height may create opportunities for certain cricket shots to emerge, while the opportunity to play other shots declines (Chow et al., 2005; Pinder et al., 2012). Expert cricket coaches have stated that cricket batters needed to be constantly attuned (e.g., perceive and use key sources of information) (Davids et al., 2012) to the slow but continuous state of change in pitch conditions, in order to score runs and minimize the chance of being dismissed (Connor, 2018). Therefore, the integrity of the pitch as game time progresses is thought to be a potentially important factor influencing cricket batting performance.

The majority of cricket batting research to date has explored skill level differences in batting technique (Stuelcken et al., 2005; Portus and Farrow, 2011; Penn and Spratford, 2012), visual anticipatory information (Renshaw and Fairweather, 2000; Müller and Abernethy, 2006), physiological demands (Scanlan et al., 2016), and the role of the individual and environment in shaping emergent behaviors (Renshaw, 2010; Connor et al., 2018). However, there has been far less analysis of performance *in situ*. Greater understanding of this area would allow for the identification of specific constraints that influence performance, and subsequently, allow practitioners and coaches to produce representative practice environments. Newell (1986) described the individual (e.g., cognitions, physical characteristics), task (e.g., equipment or implements, rules, and goals of the task) and environment (e.g., physical and socio-cultural factors) as being three critical constraints which shape emergent behavior (Higgins, 1977; Renshaw et al., 2010). For example, constraints in the form of individual cognitions play an integral role in coordinative behavior. Sarpeshkar et al. (2017b) investigated the influence of ball-swing on cricket batting performance and reported that the presence of ball swing alone resulted in altering batter’s movement timings. That is, external factors within the environment, such as ball-swing, impact a batter’s coordinative actions. Understanding what constraints impact on player’s decision-making behavior and performance is critical to creating practice environments that represent the demands of the game (Pinder et al., 2011; Barris et al., 2013).

Cricket is a unique sport in that international level teams travel and play opposition teams all around the world where different types of cricket balls and pitch or surface conditions are used depending on the home team. While there is evidence that home teams have a performance advantage because of their familiarity with these constraints (Morley and Thomas, 2005), the impact of ball type or first or second innings (an indirect marker of pitch surface deterioration) on cricket batting performance during actual game-play has yet to be examined. Therefore, the aim of this study was to investigate whether cricket batting performance is altered by the type of cricket ball used during games, and across different innings, in sub-elite, Australian cricket competition. It was hypothesized that cricket batting performance measures such as runs scored, balls faced, boundaries scored and score categories would be adversely affected during games where the Duke™ cricket ball was utilized. Further, batting performance was worse during the second innings of games, likely due to deterioration in pitch conditions. A secondary aim was to examine whether changes in ball type also impacted the mode of batter’s dismissal. Pace bowlers were hypothesized to take more wickets with the Duke™ ball compared to the Kookaburra™ based upon reported properties of the Duke™ ball.

## Materials and Methods

Game performance indicators from a total of 60 games during the 2016/17 and 2017/18 Australian Sheffield shield seasons were extracted from a commercially accessible source (http://www.espncricinfo.com; accessed from February 22nd, 2018). The analysis included 43 cricket players classified as specialist batters (i.e., listed within the first seven positions on the team list; inclusive of players, who also specialized in other roles such as wicketkeeper and all-rounders) and who played a minimum of three innings across all four experimental conditions (first innings, second innings, KB™ and Duke™ balls). The average number of games played by each batter analyzed was 15.4 ± 4.2 and were played across 15 venues around Australia. The KB™ cricket ball type was used during the first half of each season, before being switched for Duke™ balls, which were used for the second half of both seasons. All research procedures were approved by the James Cook University Human Research Ethics Committee.

### Data Analysis

Batting performance measures included average number of runs scored per inning (per dismissal), average number of balls faced, average number of boundary 4s and boundary 6s hit per game, and average strike rate (runs scored per ball and per game). Batting scores were also grouped into number of zero scores, scores of 10–24, 25–49, 50–99 and scores of 100 or more (i.e., centuries) as a percentage of the total innings included in the analyses. The relative number of wickets taken by three modes of dismissal (pace bowler, spin bowler, or run out) was also recorded as a percentage of total wickets per game.

### Statistical Analysis

Statistical analysis was conducted using the Statistical Package for Social Sciences (SPSS, version 24, IBM, IL, USA). A two-way repeated (ball type × innings) measures analysis of variance (ANOVA) was conducted on all cricket batting performance measures including runs scored, number of balls faced, average number of boundary 4 and 6s per game, and average strike rate. Finally, a three-way repeated measures ANOVA (wickets by bowler × ball type × innings) was used to compare the total relative number of wickets taken by pace bowlers, spin bowlers, or run outs when the KB™ and Duke™ cricket ball was utilized (expressed as a percentage of total games played) across both innings. For *post hoc* analyses, pairwise comparisons with Bonferroni correction were used for any significant main effects. Cohen’s D effect sizes (ES) were also computed to determine the magnitude of differences for performance measures between experimental conditions (i.e., ball type, innings). For ES calculations, 0.2 was considered as a small difference, 0.5 as a moderate difference and ≥ 0.8 as a large difference (Cohen, 1992). Statistical significance for all analyses was set at 0.05.

## Results

### Batting Performance

No significant interactions were found between innings and ball type for average runs scored [*F*(1, 42) = 0.02, *p* = 0.90], average balls faced [*F*(1, 42) = 0.26, *p* = 0.61], boundary 4s [*F*(1, 42) = 0.08, *p* = 0.78], boundary 6s [*F*(1, 42) = 0.002, *p* = 0.96], and strike rate [*F*(1, 42) = 0.25, *p* = 0.62; Table 1]. Main effects of innings demonstrated that the first innings had greater average runs scored [*F*(1, 42) = 5.13, *p* < 0.05; 37.5 ± 13.4 vs. 31.2 ± 11.3; ES = 0.45] average balls faced [*F*(1, 42) = 8.00, *p* < 0.05; 60.7 ± 26.2 vs. 49.9 ± 23.6; ES = 0.43], boundary 4s [*F*(1, 42) = 13.23, *p* < 0.01; 3.8 ± 1.9 vs. 2.9 ± 1.4; ES = 0.52], and boundary 6s [*F*(1, 42) = 10.28, *p* < 0.05; 0.2 ± 0.3 vs. 0.1 ± 0.3; ES = 0.33] compared to the second innings. No difference of innings were found for strike rate [*F*(1, 42) = 0.03, *p* = 0.86; 45.9 ± 15.9 vs. 46.3 ± 17.8; ES = 0.02].

**Table 1:** Cricket batting performance measures during each innings and for each ball type (mean ± SD).

		Runs scored	Balls faced	Boundary 4s	Boundary 6s	Strike rate
Innings 1	KB^™^	38.7 ± 17.6	60.0 ± 23.6	3.8 ± 2.0	0.2 ± 0.3	44.5 ± 10.6
	Duke^™^	36.3 ± 20.2	61.3 ± 28.9	3.8 ± 1.9	0.2 ± 0.3	47.3 ± 19.9
Innings 2	KB^™^	31.9 ± 17.2	51.2 ± 24.7	2.8 ± 1.5	0.1 ± 0.2	43.7 ± 13.5
	Duke^™^	32.0 ± 19.1	48.7 ± 22.6	3.0 ± 1.4	0.1 ± 0.3	48.8 ± 21.2

Finally, no main effect of ball type between KB™ and Duke™ was found for any variable, including average runs scored [*F*(1, 42) = 0.17, *p* = 0.70; 34.1 ± 13.5 vs. 35.3 ± 13.0; ES = 0.09], average balls faced [*F*(1, 42) = 0.02, *p* = 0.88; 55.6 ± 24.4 vs. 55.0 ± 26.6; ES = 0.02], boundary 4s [*F*(1, 42) = 0.13, *p* = 0.73; 3.3 ± 1.8 vs. 3.4 ± 1.7; ES = 0.04], boundary 6s [*F*(1, 42) = 10.28, *p* < 0.05; 0.2 ± 0.2 vs. 0.2 ± 0.3; ES = 0.07] or strike rate [*F*(1, 42) = 2.32, *p* = 0.14; 44.1 ± 12.1 vs. 48.0 ± 20.4; ES = 0.24].

### Batting Scores

No significant interaction were found between innings and ball type for scores of zero [*F*(1, 42) = 0.62, *p* = 0.44], scores of 10–24 [*F*(1, 42) = 0.58, *p* = 0.45], scores of 25–49 [*F*(1, 42) = 0.23, *p* = 0.64], scores of 50–99 [*F*(1, 42) = 0.7, *p* = 0.80], or scores of 100 of more [*F*(1, 42) = 0.47, *p* = 0.50; Table 2]. The only main effect found for score type was innings, which showed that the first innings had greater average scores of 100 or more runs [*F*(1, 42) = 15.45, *p* < 0.01; 5.74 ± 8.56 vs. 1.49 ± 5.14%; ES = 0.62] than the second innings. No main effect of innings was found for scores of zero [*F*(1, 42) = 0.43, *p* = 0.52; 8.91 ± 12.67 vs. 10.45 ± 13.93%; ES = 0.12], scores of 10–24 [*F*(1, 42) = 0.18, *p* = 0.67; 50.03 ± 20.95 vs. 51.45 ± 23.84%; ES = 0.06], scores of 25–49 [*F*(1, 42) = 1.70, *p* = 0.20; 19.31 ± 15.09 vs. 22.77 ± 18.56%; ES = 0.21] and scores of 50–99 [*F*(1, 42) = 0.89, *p* = 0.35; 5.81 ± 23.54 vs. 13.88 ± 13.96%; ES = 0.43].

**Table 2:** Percentage of games where batters scored within a certain scoring category across innings and ball type (mean ± SD).

Scores		0	10–24	25–49	50–99	100+
First innings	KB^™^	8.0% ± 10.6	52.1% ± 23.0	18.0% ± 15.6	4.7% ± 21.3	6.4% ± 7.8
	Duke^™^	9.8% ± 14.5	48.0% ± 18.7	20.6% ± 14.6	7.0% ± 25.8	5.1% ± 9.3
Second innings	KB^™^	11.2% ± 13.7	50.7% ± 21.5	22.9% ± 18.0	14.0% ± 13.7	1.3% ± 5.5
	Duke^™^	9.7% ± 14.3	52.2% ± 26.2	22.7% ± 19.3	13.7% ± 14.4	1.7% ± 4.8

No main effect of ball type between KB™ and Duke™ ball was found for scores of zero [*F*(1, 42) = 0.01, *p* = 0.93; 9.60 ± 12.30 vs. 9.76 ± 14.30%; ES = 0.01], scores of 10–24 [*F*(1, 42) = 0.15, *p* = 0.70; 51.40 ± 22.15 vs. 50.09 ± 22.73%; ES = 0.06], scores of 25–49 [*F*(1, 42) = 0.29, *p* = 0.59; 20.45 ± 16.93 vs. 21.63 ± 17.07; ES = 0.07] scores of 50–99 [*F*(1, 42) = 0.02, *p* = 0.88; 9.34 ± 18.42 vs. 10.36 ± 21.03%; ES = 0.07], or scores of 100 or more [*F*(1, 42) = 0.14, *p* = 0.71; 3.83 ± 7.16 vs. 3.41 ± 7.58%; ES = 0.06].

### Batting Dismissals

**Figure 1:**
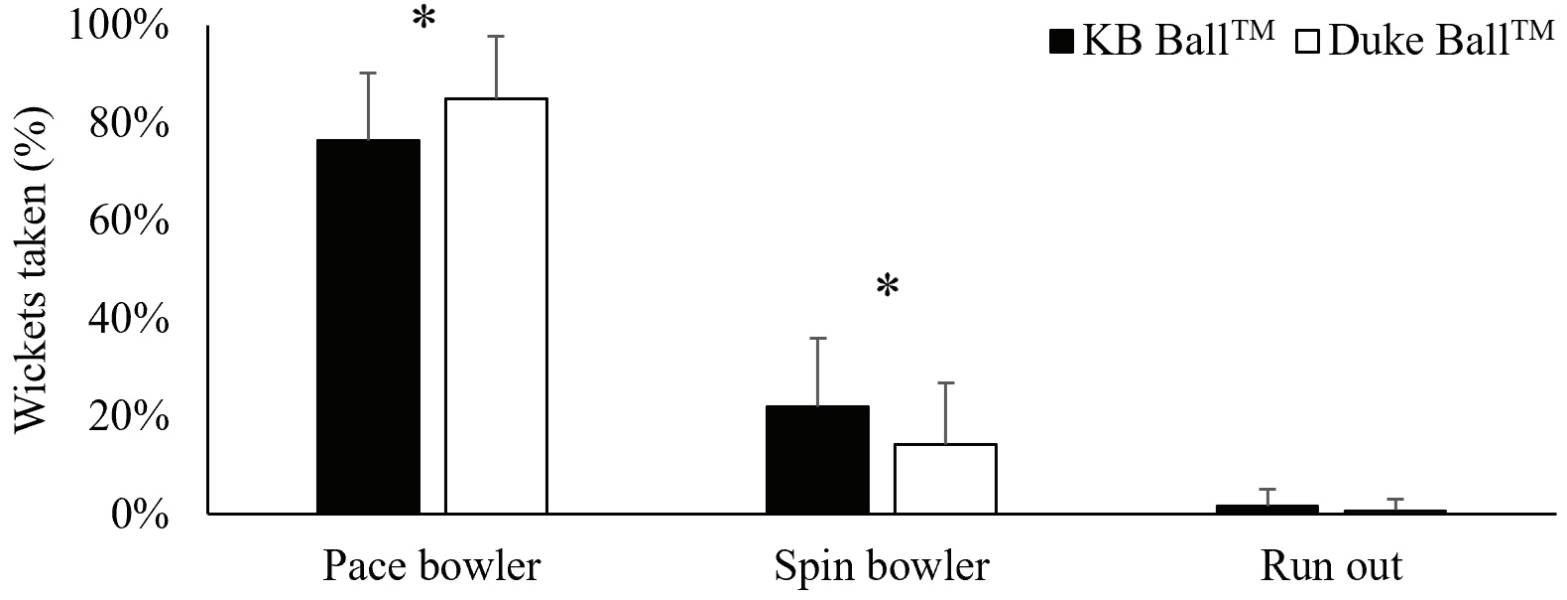
Relative number of wickets taken by either an opposition pace bowler, spin bowler, or run out. ^*^Significant difference between ball types (*p* < 0.05).

No interaction was reported between wicket taken by bowler, ball and innings [*F*(2, 84) = 0.64, *p* = 0.53]. Similarly, no interaction was found between wickets taken by bowler and innings (first innings vs. second innings: pace = 80.3 ± 11.1 vs. 82.1 ± 14.4, ES = 0.14; spin = 18.5 ± 10.6 vs. 16.7 ± 14.1, ES = 0.14; run outs = 1.2 ± 2.9 vs. 1.1 ± 3.6, ES = 0.02) [*F*(2, 84) = 0.55, *p* = 0.58]. An interaction was found between ball type and mode of dismissal [*F*(2, 84) = 7.60, *p* < 0.01]. *Post hoc* analysis revealed that a greater proportion of wickets were taken by pace bowlers with the Duke™ ball (ES = 0.72), and by spin bowlers with the KB™ ball (ES = 0.59) (Figure 1). No difference between ball types was found for run out (ES = 0.27).

## Discussion

The aims of this study were to examine: (1) the impact of evolving constraints, specifically innings and ball type, on batting performance; and (2) whether batter’s dismissal by pace or spin bowlers was affected by innings or ball type. Ball type had no impact upon batting performance, whereas greater batting performance was identified during the first innings compared to the second innings. Further, ball type influenced the relative number of wickets taken by bowlers with pace bowlers taking more wickets with the Duke™ ball while spin bowlers took more wickets with the KB™ ball. These findings suggest that state level cricket batters transferred batting performance from the KB™ ball to the Duke™ ball; however, did not transfer their skills as effectively to the changing constraints associated with second inning’s performances. Given pace bowlers took a greater proportion of wickets with the Duke™ ball, differences between the two ball types may exist with future studies needed to confirm. These findings are important to coaches and players when preparing for an upcoming game, whereby the batter’s preparation and subsequent game strategy should be individualized to the game constraints.

Interestingly, no differences were found in batting performance measures during games that used either the KB™ or Duke™ ball. The overall findings with respect to ball type were somewhat surprising given anecdotal reports of players, and previous empirical work, highlighting the increased challenge of batters in intercepting a swinging ball (Sarpeshkar and Mann, 2011; Sarpeshkar et al., 2017a,b). The properties of the Duke™ ball have been purported to induce greater swing and for a longer duration (Alam et al., 2007; Rundell, 2009), however this has not been established empirically in cricket. The current study identified that the relative scoring distribution during KB™ and Duke™ ball games were minor as evident by small ES, suggesting that any potential changes in the ball flight trajectory as a result of the Duke™ ball’s innate properties did not impact upon batting performance measures. That is, batters successfully transferred their batting skill to the alternate ball conditions. Alternatively, batters’ may not have needed to change behavior due to a lack of clear difference between the Duke™ and KB™ ball’s ability to swing to a greater extent. Further empirical clarification is required in this area.

Regardless of the type of cricket ball used, the significant impact of innings on cricket batting performance measures was in line with previous findings (Borooah and Mangan, 2010). Specifically, Borooah and Mangan (2010) analyzed top performing cricket batters and reported that 80% of the top 50, highest batting average players scored more runs during the first innings than the second innings. In the current study, batting performance (i.e., runs scored, balls faced, boundaries 4s and 6s, and centuries scored) were all significantly lower during the second innings with a moderate effect size identified for boundary 4s. Previous research has shown that successful batting performance and run scoring is associated with greater quality of bat-ball contact (Connor et al., 2018). However, the majority of research to date on cricket batting has been conducted under controlled settings with limited discussion on the state of the playing surface. Cricket pitches often deform when repeatedly impacted by external factors (e.g., cricketer’s running on the pitch, bowling deliveries, etc.), influencing the consistency of the ball trajectory (Carré et al., 1999; Carré and Haake, 2000) and can create greater spatio-temporal demands of batters trying to achieve bat-ball contact. Carré et al. (1999) reported changes in ball velocity and rebound angle (i.e., bounce) when comparing the pitch surface properties from day one to day four, a similar time period of sub-elite cricket games. Therefore, one possible explanation for the current results may be that batters achieved better bat-ball contact quality during the first innings, where the pitch surface had yet to deteriorate, which reduced the opportunities for the opposition bowler to bring about a dismissal. However, further work which includes mechanistic data (e.g., bat-ball contact quality, shot type, etc.) is required to support this explanation. Further research is also recommended to examine whether the speed/pace, bounce and/or consistency properties of the pitch influence cricket batting behavior or performance.

Another substantial difference between first and second innings is the overall situation of the game, and thus, the roles and responsibilities of the batter (Scarf et al., 2011). The second innings require batters to score a sufficient number of runs, such that the opposition cannot (1) score as many or (2) bat for a duration that takes the game to a draw if the team is unable to win. Akhtar and Scarf (2012) highlighted the complexity of second innings’ performances, whereby a 10% increase in required runs beyond a critical point can result in a 20% reduction in win probability. Reductions in second inning’s performance may result from an increase in perceptual and technical demands, thought to be associated with a deteriorating pitch surface and spontaneous changes in ball trajectory, as well as the greater cognitive demands from an evolving penultimate game situation leading to the final game outcome. It is important to note that within this study, there was no difference in strike rate (i.e., runs scored per hundred balls) identified between first and second innings’ performances, suggesting that the game scenario demands did not differ enough to alter the speed in which runs needed to be scored between innings. It is recommended that coaches create practice environments that both mimic the playing surface that commonly occurs during second innings (e.g., 3- or 4-day old pitch surfaces) coupled with scenarios that batters are likely to experience during second inning’s performances.

Finally, scores of ≥100 (5.74 vs. 1.49%, moderate ES) occurred significantly more during the first innings when compared with the second innings. Effect sizes for all other score comparisons (0, 1–24, 25–49, and 50–99) were small. It is suggested that the observed differences in average runs scored between innings occur in part due to the batter’s inability to convert scores of 50–99 (5.81 ± 23.54 vs. 13.88 ± 13.96%) into 100 or more during the second innings. By this late stage of the game, both physical (Scanlan et al., 2016; Cooke et al., 2018) and mental (Veness et al., 2017) fatigue are likely to be contributing factors to performance. It has also been previously reported that batters experience heightened anxiety when achieving important milestones (e.g., scoring 100 or more runs) and during the initial period of batting (Slogrove et al., 2002).The aforementioned pitch deterioration is likely to be another significant factor on batting performance (Carré et al., 1999). Together, these greater individual and environmental demands upon the batter, occurring in the latter part of the second innings, may explain the decline in performance. To counteract these performance decrements, it is recommended that coaches and practitioners ensure players are exposed to conditions that they are likely to experience during a game, such as physical and mental fatigue, anxiety and pitch deterioration (e.g., uneven ball bounce and pace). Following a representative learning design approach would ensure players maintain both action fidelity and functionality during their training (Pinder et al., 2011).

Interestingly, despite no impact of ball type on batting performance, opposition pace bowlers took relatively more wickets with the Duke™ ball than during games involving the KB™ ball. The greater bowling success of pace bowlers with the Duke™ ball may be a result of the opposition team exploiting the propensity of Duke™ balls to swing more, and thus, predominantly utilize pace bowlers during the game rather than spinners. Additionally, fast bowler’s may also adapt their bowling technique (e.g., keeping the seam more upright) to provide the greatest opportunity for the ball to swing. While a swinging ball perturbs batting technique and perceptual demands (Sarpeshkar and Mann, 2011; Sarpeshkar et al., 2017b), which can result in reduced proficiency of an interceptive task (Craig et al., 2006), the current study demonstrates that batting performance was not affected as a result of the type of cricket ball. While it is not known exactly how batters were able to effectively adapt to different ball types, one explanation may be the logistical ease in which coaches can incorporate different cricket balls into their practice environments. By doing so, players can calibrate their actions to any potential altered ball trajectory associated with various ball types. Future research should examine the duration of time required for batters to effectively transfer skills to various ball types used in cricket games, so as to provide evidence-based coaching recommendations for match preparation during the preseason.

A limitation inherent in this study was the order effect in which batters were exposed to different cricket ball conditions. Across both seasons, the first half of the season utilized the KB™ ball while the latter half utilized the Duke™. Ideally, comparing both ball conditions would be conducted with a randomized cross-over design to enable examination of the transfer of skills from the Duke™ to KB™ ball, and from the KB™ to the Duke™ ball. However, the real-world collection of data meant that this was not feasible. It is therefore unclear whether the preparation time between the first and second half of the season was a primary factor for batting performance being relatively unaffected by the use of a different cricket ball. It was also unclear as to whether different pitch surfaces deteriorated more quickly than others during games, which should be a factor considered in future research. Analytical approaches may have also impacted the current outcomes; although this was unlikely given that additional analyses on individual case data were conducted using linear mixed modeling, to consider the variance across individual performances, with similar outcomes produced. Cricket batting performance may also be best analyzed in conjunction with additional performance indicators and mechanistic measures of cricket batting (e.g., types and efficiency of shots executed). This is due to performance being dependent on a multitude of factors that exist within the use of different ball types or pitch surfaces. For example, a number of individual (e.g., emotions, motivations, intentions) and task constraints (e.g., situation of the game, field-settings) are influential factors that need to be considered and standardized to an extent between participants. While cricket batting performance measures are crucial to understanding the real-world effect, future research should investigate the impact of ball type, and the constraints associated with different innings, following a representative approach under controlled conditions.

## Conclusions

The current study demonstrated that cricket batting performance was comparable in games involving the KB™ and a Duke™ ball, despite pace bowlers being more successful with a Duke™ ball and spin bowlers more successful with the KB™ ball. Therefore, state level cricket batters were able to transfer their batting performance from the KB™ ball to the Duke™ ball condition. Further, batters were not able to commensurately adapt to the constraints concomitant with second innings of the game, with batting performance significantly less during the second innings regardless of ball type. Deteriorating pitch surfaces and the mutable role of the batter during the second innings, which may result in a shift in intentions and batting technique, may partly explain the decline in performance. Future research could identify how batting technique and actions differ between innings and allow coaches to create practice environments that are representative of the demands of the game. The practical applications of this study can be used by coaches to incorporate second innings’ conditions in their training practices, similar to the way different ball types are utilized during practice. Logistically, it may only be possible to implement this over a significant period of time during pre-season preparation. This learning design would provide batters with the opportunity to adapt their own individual game and movement-specific strategies in response to the real demands experienced during the second innings of cricket matches.

## Data Availability Statement

The datasets generated for this study are available on request to the corresponding author.

## Ethics Statement

All research procedures were approved by the James Cook University Ethics Committee. All data utilized in this study was accessed from an opensource website (www.espncricinfo.com) which displayed the game performance scores of each individual player. Therefore, consent of individual participants was not required.

## Author Contributions

All authors contributed ideas to the design of this study. JC performed the statistical analyses. JC developed the first paper draft, and all authors revised the manuscript for important intellectual content and approved the final version of the article.

### Conflict of Interest

The authors declare that the research was conducted in the absence of any commercial or financial relationships that could be construed as a potential conflict of interest.
